# Circadian-guided oncolytic virotherapy for glioblastoma

**DOI:** 10.1007/s13365-026-01325-7

**Published:** 2026-07-06

**Authors:** Alexandro Guterres

**Affiliations:** Department of Genetics, Ribeirão Preto Medical School, University of São Paulo – USP, Av. Bandeirantes, 3900 – Monte Alegre Ribeirão Preto, São Paulo, 14049-900 SP Brazil

**Keywords:** Glioblastoma, Circadian Rhythms, Oncolytic Viruses, Chronotherapy, Precision Immunotherapy

## Abstract

Glioblastoma (GBM) remains one of the most aggressive and lethal malignancies, with clinical outcomes under the standard-of-care Stupp protocol remaining suboptimal. While oncolytic virus (OV) therapy has emerged as a mechanistically distinct and promising approach, its clinical success is frequently hindered by profound intratumoral heterogeneity and emerging resistance. This critical review proposes that the efficacy of virotherapy may be meaningfully enhanced by integrating circadian biology into treatment schedules; a paradigm termed “Oncolytic Chronovirotherapy.” Recent evidence demonstrates that the infection cycle of neurotropic viruses, which serve as the scaffold for many OVs, is not a static process but is inherently regulated by the host’s circadian clock. Fundamental studies reveal that the expression of essential viral entry receptors, such as Nectin-1 (for HSV-1) and p75NTR, exhibits robust circadian oscillations directly coordinated by the core molecular clock, specifically the BMAL1/CLOCK complex. Furthermore, the tumor immune microenvironment and cellular vulnerability to adjuvant chemotherapy (temozolomide) show synchronized peaks of activity, typically concentrated during the morning window, which often coincides with the nadir of DNA repair enzymes like MGMT. We argue that synchronizing OV administration with these windows of maximal receptor density and robust immune surveillance may maximize tumor lysis and facilitate the conversion of “cold” tumors into “hot,” immune-responsive environments. The utilization of personalized circadian biomarkers and mathematical modeling to guide therapy delivery will be essential for the next generation of precision neuro-oncology.

## Introduction

Glioblastoma (GBM) stands as the most aggressive primary malignancy of the central nervous system, with a prognosis that has remained largely refractory to medical advances for decades. The current standard of care, maximal safe surgical resection followed by the Stupp protocol (radiotherapy and temozolomide), offers a median overall survival of only 15 to 20 months (Stupp et al. 2015). This therapeutic failure is driven by GBM’s profound intratumoral heterogeneity, rapid acquisition of resistance, and a “cold” tumor microenvironment (TME) characterized by low T-cell infiltration and a dominant immunosuppressive landscape of M2-like macrophages and myeloid-derived suppressor cells (Thakur et al. 2022; Sharma et al. 2023). In this context, conventional immunotherapies, such as immune checkpoint inhibitors (ICIs), have largely failed to produce durable clinical benefits in GBM patients (Ling et al. 2023).

Oncolytic virus (OV) therapy has emerged as a mechanistically distinct paradigm designed to bypass these hurdles (Tian et al. 2022). Engineered viruses, such as the Japanese-approved HSV-1 G47Δ, are designed to selectively replicate within and lyse malignant cells, releasing tumor-associated antigens (TAAs) and damage-associated molecular patterns (DAMPs) (Todo et al. 2022). This process serves as an in situ vaccination, potentially converting the “cold,” immunosuppressed GBM milieu into a “hot,” immune-responsive environment by recruiting cytotoxic T lymphocytes and reprogramming local innate immunity (Nair et al. 2021). Despite this potential, clinical responses to OVs remain inconsistent, often limited by individual variations in viral entry, replication, and the host’s fluctuating immune state (Lipatova et al. 2021).

A critical, yet overlooked, factor in this variability is the role of the circadian clock, the endogenous 24-hour timing system that orchestrates nearly all aspects of cellular physiology and immune function (Lowrey and Takahashi 2004). In GBM, the molecular clock is frequently dysregulated to favor tumor progression and stemness, yet it retains rhythmic control over critical nodes such as DNA repair enzymes (e.g., MGMT) and viral entry receptors like Nectin-1 (De La Cruz Minyety et al. 2021; Wan et al. 2025; Gonzalez-Aponte et al. 2026). Furthermore, systemic immune surveillance is not static; leukocyte trafficking, dendritic cell maturation, and T-cell effector functions exhibit robust circadian oscillations that dictate the efficacy of both vaccinations and immunotherapies (Qian et al. 2021; Özdemir et al. 2025; Liu et al. 2026).

This critical review proposes a paradigm shift toward “Oncolytic Chronovirotherapy.” We argue that the efficacy of viral oncolysis and subsequent immune activation is inherently time-dependent. By integrating circadian profiling and mathematical modeling into treatment schedules, we can exploit specific windows of maximal tumor vulnerability and peak immune competence. Transitioning from a static treatment model to a dynamic, time-aware strategy represents an essential frontier in precision neuro-oncology, offering a potential breakthrough to overcome the formidable resistance of glioblastoma.

## The dysregulated molecular clock in glioblastoma

### The canonical circadian architecture

The mammalian circadian clock is governed by an evolutionarily conserved, cell-autonomous system of interlocking transcriptional-translational feedback loops (TTFLs) (Badiu 2003; Li et al. 2006). At the core of the primary loop, the basic helix-loop-helix transcription factors CLOCK (Circadian Locomotor Output Cycles Kaput) and BMAL1 (Brain and Muscle ARNT-like 1, encoded by *ARNTL*) heterodimerize in the cytoplasm. This complex translocates into the nucleus to bind enhancer box (E-box) elements in the promoter regions of the Period (*PER1*, *PER2*, *PER3*) and Cryptochrome (*CRY1*, *CRY2*) genes, driving their rhythmic expression. As PER and CRY proteins accumulate, they form repressive complexes that feedback into the nucleus to inhibit CLOCK: BMAL1 activity, effectively closing the 24-hour cycle (Takahashi 2017; Cox and Takahashi 2019). This primary oscillator is further refined by a secondary loop where CLOCK: BMAL1 activates the transcription of nuclear receptors REV-ERBα/β (repressors) and RORα/β/γ (activators), which compete for ROR-binding elements (RORE) on the BMAL1 promoter, ensuring high-amplitude and robust oscillations (Takahashi 2017).

### Hijacking the positive limb: CLOCK and BMAL1 as oncogenic drivers

In the context of glioblastoma, this temporal harmony is systematically dismantled to favor malignancy. The “positive limb” genes, *CLOCK* and *BMAL1*, are consistently reported as overexpressed in high-grade gliomas compared to normal brain tissue (Chen et al. 2013; Huang et al. 2019; Arafa and Emara 2020; Zhang et al. 2021). Rather than merely oscillating, these factors act as oncogenic drivers that facilitate tumor proliferation and survival. Specifically, *CLOCK* overexpression has been shown to enhance the expression of cyclins A, B1, and D1 through its intrinsic histone acetyltransferase activity, while simultaneously activating the NF-κB pathway (Nelson and Relógio 2024). Crucially, the survival of glioblastoma stem cells (GSCs), the highly resistant subpopulation responsible for recurrence, is uniquely dependent on the BMAL1/CLOCK complex (Albaqami 2025; Nettnin et al. 2025). Experimental depletion of these genes has been shown to induce p53-dependent apoptosis and impair the stemness of GSCs, while leaving normal neural stem cells relatively unaffected (Nelson and Relógio 2024; El-Tanani et al. 2024). Furthermore, the CLOCK: BMAL1 complex directly orchestrates metabolic reprogramming in GBM, driving the Warburg effect by transcriptionally activating key glycolytic enzymes like LDHA and HK2, and facilitating de novo lipid synthesis to support rapid membrane biogenesis (Nelson and Relógio 2024; El-Tanani et al. 2024; Zhang et al. 2026).

### Suppression of the negative limb and the loss of tumor control

Conversely, the “negative limb” of the clock, comprising the PER and CRY families, often functions as a tumor-suppressive axis that is repressed in adult GBM. Low expression levels of PER1, PER2, and PER3 are strongly associated with advanced tumor grade and poorer overall survival. PER2, in particular, acts as a critical node connecting the clock to the DNA damage response; it binds to and stabilizes the tumor suppressor p53, preventing its MDM2-mediated proteasomal degradation (Zhang et al. 2021; De La Cruz Minyety et al. 2021; Petkovic et al. 2023; Nelson and Relógio 2024). The downregulation of PER genes in GBM thus facilitates the evasion of apoptosis and the accumulation of genomic instability. Similarly, CRY2 is frequently suppressed in GBM, and its loss correlates with the hyperactivation of oncogenic signaling pathways such as c-Myc, which is normally targeted for degradation by CRY2 in cooperation with the SCF-FBXL3 ubiquitin ligase complex (Nelson and Relógio 2024; Albaqami 2025; Zhang et al. 2026).

Collectively, the dysregulation of the molecular clock in GBM exerts a pervasive and coordinated influence across all major axes of tumor biology relevant to therapeutic resistance. Cell cycle progression is directly gated by BMAL1/CLOCK-dependent transcription of WEE1, Cyclin B1, and CDK1; constitutive deregulation of this gate removes a critical mitotic checkpoint and fuels unrestrained proliferation (Fagiani et al. 2022; Amiama-Roig et al. 2022). DNA repair fidelity is compromised not only through oscillation of MGMT activity (detailed in Sect.  5) but also through the role of PER2, which beyond p53 stabilization, participates in nucleotide excision repair scaffolding in coordination with XPA; its suppression in GBM thus simultaneously dismantles apoptotic and repair checkpoints, enabling genomic instability (Kang and Sancar 2009; Sancar and Van Gelder 2021; Amiama-Roig et al. 2022). Apoptotic sensitivity is further undermined by CRY2 loss and consequent hyperactivation of c-Myc and the evasion of SCF-FBXL3-mediated proteasomal degradation. Metabolic reprogramming toward aerobic glycolysis is actively orchestrated by the CLOCK: BMAL1 complex through transcriptional activation of LDHA and HK2, alongside facilitation of de novo lipid synthesis for membrane biogenesis (Huber et al. 2016; Sulli et al. 2019; Petkovic et al. 2023; Zhang et al. 2026). Finally, tumor invasion is temporally regulated through circadian modulation of matrix metalloproteinase (MMP) expression and epithelial-mesenchymal plasticity, which oscillate in clock-dependent fashion in glioma models (JUNG et al. 2013; Petkovic et al. 2023). This comprehensive circadian control over the full spectrum of GBM hallmarks underscores that therapeutic timing is not merely a pharmacological refinement but a fundamental dimension of tumor vulnerability, the strategic exploitation of which is the central argument of this review.

### Circadian modulation of the tumor microenvironment (TME)

The dysregulation of the molecular clock extends beyond the neoplastic cell, actively shaping the immunosuppressive TME (El-Tanani et al. 2024; Nagy et al. 2025; Zhang et al. 2026; Liu et al. 2026). The overexpressed CLOCK: BMAL1 complex in GBM cells promotes the recruitment of M2-like tumor-associated macrophages (TAMs) and myeloid-derived suppressor cells (MDSCs) through the activation of the OLFML3-HIF1α axis (Nelson and Relógio 2024; Albaqami 2025). This interaction not only fuels tumor angiogenesis but also creates a “cold” immune landscape characterized by T-cell exhaustion and elevated PD-L1 expression (Nelson and Relógio 2024; Wan et al. 2025; Liu et al. 2026). Interestingly, this crosstalk is reciprocal: TAMs can secrete exosomes carrying microRNAs (such as miR-7239-3p) that further reprogram the tumor’s clock, creating a self-reinforcing loop of temporal disruption and immune evasion (Nelson and Relógio 2024). This profound circadian plasticity underscores the necessity of a time-aware therapeutic intervention to disrupt the temporal foundations of glioblastoma progression.

## Rhythm-dependent viral infectivity

### The circadian gating of viral entry receptors

**Fig. 1:**
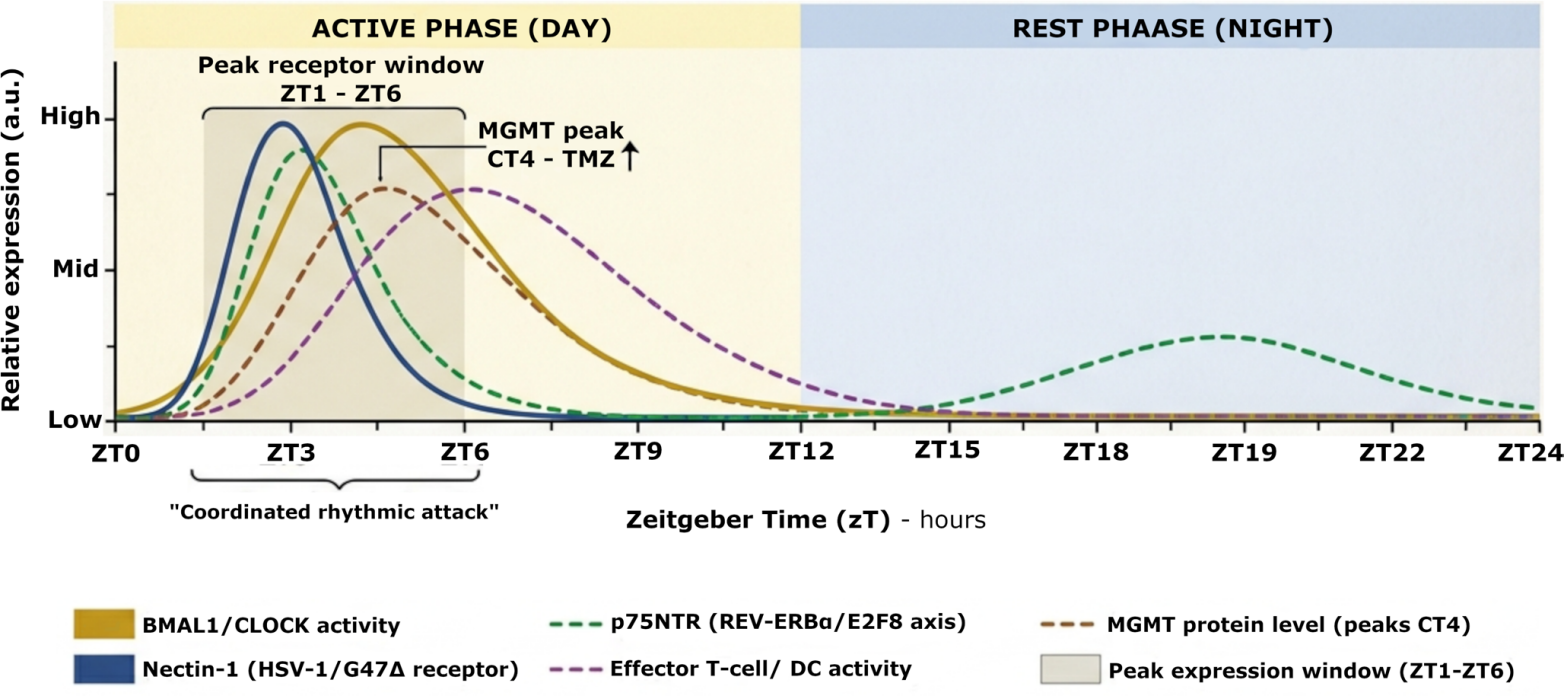
Circadian oscillations of viral entry receptors across the 24-hour cycle. The core clock protein BMAL1 drives rhythmic expression of Nectin-1 (the primary receptor for HSV-1–based oncolytic viruses such as G47Δ) and p75NTR (gated via the REV-ERBα/E2F8 axis). These receptors exhibit robust oscillations peaking during the morning window (ZT1–ZT6 in diurnally active humans). This peak aligns with maximal cellular susceptibility to oncolytic infection and coincides with the window of peak effector immune function and dendritic cell alertness. Although protein levels of the repair enzyme MGMT also peak in the morning (CT4, or 4 hours into the internal circadian cycle), mathematical models confirm that TMZ efficacy is maximized when administered during this phase, creating a ‘coordinated rhythmic attack’ for Oncolytic Chronovirotherapy. ZT, Zeitgeber Time; CT, Circadian Time (internal biological time in synchronized conditions); a.u., arbitrary units

The first and most critical step in the viral life cycle, the engagement of the virion with host cell surface receptors, is not a static event but is profoundly regulated by the host’s internal timing (Borrmann et al. 2021; Zhuang et al. 2022). Recent high-throughput analyses using Cleavage Under Targets and Tagmentation (CUT&Tag) in human cerebral organoids have revealed that the core clock protein BMAL1 exhibits distinct binding peaks in the promoter regions of at least 32 neurotropic virus receptors This includes essential entry nodes for major viral scaffolds used in oncolytic therapy, such as PDGFRA, AXL, and ITGB1 (Integrin β1) (Zeng et al. 2026). For instance, the expression of Nectin-1 (encoded by PVRL1), the primary receptor mediating the entry of Herpes Simplex Virus type 1 (HSV-1) into neurons, exhibits robust rhythmic oscillations coordinated directly by the molecular clock. Mechanistically, the BMAL1/CLOCK heterodimer binds directly to E-box-like motifs within the NECTIN-1 promoter, enhancing its transcriptional activity and creating specific daily windows of heightened cellular susceptibility(Tang et al. 2025) (Fig. 1).

### Complex regulatory circuits: The REV-ERBα and E2F8 axis

While some receptors are directly gated by the positive limb of the clock, others are regulated through sophisticated intermediate repressive circuits. A prime example is the regulation of the p75NTR receptor, used by neurotropic viruses like the rabies virus (RABV). The *p75NTR* mRNA levels oscillate in phase with the transcriptional repressor REV-ERBα (Zeng et al. 2026). This regulation is mediated by the cell cycle regulator E2F8, which acts as a transcriptional repressor of *p75NTR*. REV-ERBα rhythmically suppresses E2F8 expression, thereby periodically relieving the inhibition on *p75NTR* and facilitating viral entry. This “repressor of a repressor” mechanism demonstrates that the circadian clock’s influence on viral infectivity extends beyond simple linear pathways, involving interlocked feedback loops that dictate the temporal landscape of the cell surface (Zeng et al. 2026).

### Temporal heterogeneity in viral load and pathogenesis

Experimental evidence across multiple species confirms that the timing of infection significantly determines the outcome of viral pathogenesis. In mouse models, HSV-1 and HSV-2 infections are consistently less severe when they occur during the rest phase compared to the active phase (Borrmann et al. 2021; Zhuang et al. 2022; Tang et al. 2025). For example, the dose of antiviral medication (e.g., acyclovir) required to prevent HSV-2 infection can be up to four times higher during the active phase than during the resting phase, reflecting the clock-dependent density of Nectin-1 receptors (Matsuzawa et al. 2018; Zhuang et al. 2022). Similarly, RABV infection at ZT12 (onset of the active phase in mice) results in significantly higher viral loads and more severe encephalitis compared to infection at ZT0, a phenomenon attributed to the circadian gating of p75NTR (Zeng et al. 2026). These findings suggest that the initial viral “take” is highly sensitive to the internal biological time of the host.

### Exploiting circadian vulnerability for oncolytic virotherapy

For oncolytic viruses (OVs) like G47Δ or G207, which are engineered from neurotropic backbones like HSV-1, these circadian principles offer a transformative opportunity(Yang et al. 2025). Glioblastoma cells, and particularly glioblastoma stem cells (GSCs), often retain a functional, albeit dysregulated, molecular clock that drives the rhythmic expression of these same entry receptors (Nelson and Relógio 2024; Albaqami 2025). By synchronizing the administration of OVs with the peak expression of receptors like Nectin-1 or CD155, clinicians may maximize the initial infection rate and subsequent intratumoral spread (Yang et al. 2025; Tang et al. 2025). Furthermore, since standard treatments like temozolomide (TMZ) also show time-dependent efficacy, peaking in the morning when BMAL1 is high and the repair enzyme MGMT is at its nadir, the integration of OVs into a morning-based “chronovirotherapy” schedule could synergistically enhance both direct viral lysis and chemosensitivity (De La Cruz Minyety et al. 2021; Nelson et al. 2025; Gonzalez-Aponte et al. 2026).

Critically, the chronobiological principles outlined above are not restricted to HSV-1-based vectors and may be extended to the broader landscape of oncolytic viruses that have already demonstrated clinical proof-of-concept in GBM, as comprehensively reviewed by Coates and Collaborates (Coates et al. 2024), an oncolytic adenovirus that enters cells via αvβ3 and αvβ5 integrins through an engineered RGD-4c motif, demonstrated tumor regression in 72% of patients and survival exceeding three years in five patients in a Phase I trial of 37 recurrent GBM cases (Lang et al. 2018). Notably, ITGB1 (Integrin β1), a core subunit of these same integrin heterodimers, is among the 32 neurotropic virus entry receptors identified by CUT&Tag analyses as bearing BMAL1 transcriptional binding peaks in human cerebral organoids (Zeng et al. 2026), raising the hypothesis that the circadian gating of integrin expression may modulate adenoviral OV entry through the same molecular mechanism described for HSV-1. MV-CEA, an oncolytic measles virus that exploits CD46 overexpression on GBM cells for selective entry, achieved a median overall survival of 11.6 months and a 1-year survival rate of 45.5% in a Phase I trial (Galanis et al. 2024); the observation that treatment response correlated inversely with baseline interferon-stimulated gene (ISG) expression is particularly relevant, as IFN signaling itself exhibits circadian regulation, suggesting that the time of OV administration may interact with the circadian state of the innate antiviral response within the TME (Greenberg et al. 2020; Schäfer et al. 2024; Coates et al. 2024).

PVSRIPO, a poliovirus-rhinovirus chimera targeting the CD155 receptor, generated a durable overall survival plateau of 21% at both 24 and 36 months in a Phase I GBM trial, a long-tail pattern consistent with immune-mediated mechanisms (Stavrakaki et al. 2021; Coates et al. 2024; Liu et al. 2025). Since CD155 is already identified in our chronovirotherapy framework as a circadianly relevant viral entry receptor (Sect.  3), temporal optimization of PVSRIPO administration is directly supported by the existing mechanistic evidence. Together, these clinically validated platforms provide the essential foundation upon which to design prospective, time-of-day interventional trials across the OV field (Xu et al. 2025; Liu et al. 2025).

## Chrono-immunology and the tumor microenvironment

### The circadian landscape of systemic and local immunity

The mammalian immune system is not a static defense network but a highly dynamic system governed by the circadian timing system (CTS) (Karaboué et al. 2024). In humans, circulating counts of nearly all leukocyte subsets exhibit high-amplitude 24-hour oscillations (Karaboué et al. 2024; Huang et al. 2025). Total lymphocytes reach their trough (minimum) in the morning (08:00–10:00) and peak during the early night (00:00–02:00)(Karaboué et al. 2024). However, the efficiency of immune surveillance is often anti-phasic to these numbers; while undifferentiated T cells peak during sleep, effector T cells and Natural Killer (NK) cells, the primary mediators of anti-tumor cytotoxicity, demonstrate peak activity and infiltration potential during the morning and early afternoon hours (Amiama-Roig et al. 2022; Karaboué et al. 2024). This systemic rhythmicity is mirrored within the tumor immune microenvironment (TIME), where the infiltration of CD8 + T cells and dendritic cells (DCs) is strictly regulated by clock-controlled chemotactic signals, such as the diurnal oscillation of CCR7 and CXCR4 (Diallo et al. 2020; Karaboué et al. 2024; Nagy et al. 2025).

A key endocrine transducer of this circadian immune architecture is melatonin, synthesized and secreted by the pineal gland predominantly during the dark phase. Serum melatonin concentrations rise approximately two hours before sleep onset, peak between 02:00 and 04:00 (CT16–CT20), and decline sharply at dawn (Lévi et al. 2024; Karaboué et al. 2024). Beyond its chronobiotic function, melatonin exerts direct immunostimulatory effects via MT1 and MT2 receptors expressed on lymphocytes and NK cells: it enhances NK cell cytotoxic activity, promotes production of pro-inflammatory and antiviral cytokines (IL-2, IL-12, IFN-γ), and potentiates T-cell effector responses (Zhang et al. 2021; Munteanu et al. 2024). This nocturnal immune-priming function is not in conflict with, but rather complementary to, the morning-focused Oncolytic Chronovirotherapy window proposed in this review. The hormonal transition from peak nocturnal melatonin to rising morning cortisol supports a sequential two-phase model of circadian immune readiness: nocturnal melatonin-mediated ‘priming’ of NK and T cells during sleep, followed by cortisol-driven deployment of effector immune function and DC maturation during the morning waking hours, precisely the window proposed for OV administration (Zhou et al. 2022). Whether pharmacological melatonin supplementation at night could amplify this morning immune effector window, thereby potentiating the vaccine-like effect of OV-induced antigen release, remains an open and clinically testable hypothesis (Maitra et al. 2019; Zhang et al. 2021).

### Clock-controlled myeloid reprogramming in the GBM niche

Glioblastoma is notorious for its “cold” and profoundly immunosuppressive TME, which can comprise up to 50% tumor-associated macrophages (TAMs) and microglia (Nelson and Relógio 2024). The molecular clock in both neoplastic cells and myeloid cells plays a decisive role in maintaining this state. The CLOCK: BMAL1 complex in GBM cells directly activates the transcription of the chemokine OLFML3, which recruits microglia and promotes their reprogramming into a pro-tumorigenic, M2-like phenotype through the HIF1α/LGMN axis (Nelson and Relógio 2024; Albaqami 2025). Conversely, a deficiency in BMAL1 in TAMs increases reactive oxygen species (ROS) and skews them toward an immunosuppressive state, highlighting the necessity of clock integrity for a “hot” TME (Zhang et al. 2026). Furthermore, glioblastoma cells can distally and locally rewire these rhythms; for instance, tumor-reprogrammed microglia secrete exosomes containing miR-7239-3p, which feeds back to the tumor to downregulate BMAL1 and upregulate CLOCK, creating a self-sustaining cycle of temporal disruption and immune evasion (De La Cruz Minyety et al. 2021; Nelson and Relógio 2024).

### Temporal gating of antigen presentation and checkpoint expression

The success of oncolytic viruses (OVs) as in situ vaccines depends on the efficient presentation of tumor-associated antigens (TAAs) and the relief of immune checkpoints (Karaboué et al. 2024; Liu et al. 2025). Evidence shows that DC maturation and their migration to tumor-draining lymph nodes peak during specific daily windows, driven by BMAL1-dependent expression of CD80 (Kisamore et al. 2023; Nagy et al. 2025). In murine models, the intensity of T-cell-mediated responses to antigens is significantly higher during the daytime (subjective morning for humans), a period associated with lower inhibitory function of regulatory T cells (Tregs) (Liu et al. 2026). Crucially, the expression of immune checkpoints like PD-1 on T cells and PD-L1 on tumor cells is not constant; they exhibit robust circadian oscillations (El-Tanani et al. 2024; Huang et al. 2025; Albaqami 2025). Administering therapy during the nadir of PD-1/PD-L1 interactions, which typically occurs in the morning hours, can theoretically maximize the reinvigorating effect of the immune system and the subsequent cytotoxic spread induced by the viral infection (El-Tanani et al. 2024; Liu et al. 2026).

### Oncolytic viruses as rhythmic “Cold-to-Hot” converters

**Fig. 2:**
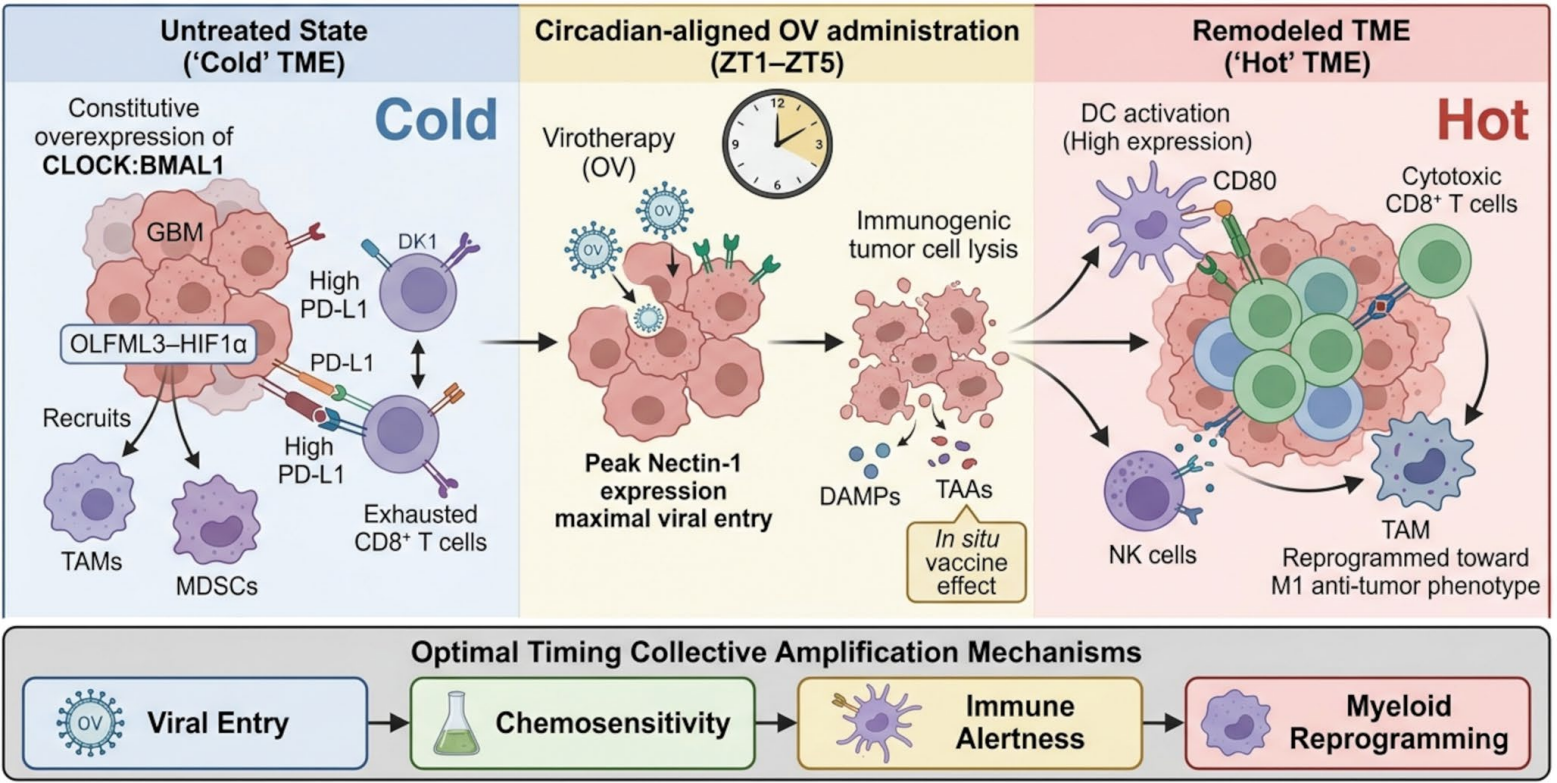
Schematic of Oncolytic Chronovirotherapy-mediated conversion of the GBM tumor immune microenvironment (TME) from ‘cold’ to ‘hot’. In the untreated state (left), GBM cells constitutively overexpressing CLOCK: BMAL1 recruit M2-like tumor-associated macrophages (TAMs) and myeloid-derived suppressor cells (MDSCs) via OLFML3–HIF1α, establishing an immunosuppressive niche characterized by exhausted CD8 + T cells and high PD-L1 expression. Circadian-aligned OV administration during ZT1–ZT5 (center) exploits peak Nectin-1 expression for maximal viral entry, inducing immunogenic tumor cell lysis and releasing DAMPs and TAAs that serve as an in situ vaccine. This triggers TME remodeling (right): dendritic cell activation (CD80↑), cytotoxic CD8 + T-cell infiltration, NK cell engagement, and TAM reprogramming toward an M1 anti-tumor phenotype. The bottom bar summarizes the four circadian mechanisms (viral entry, chemosensitivity, immune alertness, and myeloid reprogramming) that collectively amplify this response when timing is optimized

Oncolytic virotherapy, such as oHSV-1 (G47Δ), triggers immunogenic tumor cell death, leading to the release of DAMPs and TAAs that reshape the TME (Hu et al. 2023; Ling et al. 2023; Liu et al. 2025). This transformation involves the recruitment of bone marrow-derived anti-tumor lymphocytes and the distinctive activation of resident microglia(Reale et al. 2024). However, if the OV-induced inflammatory stimulus occurs during a phase of circadian immune “quiescence” (e.g., late afternoon/evening), the resulting immune recruitment may be suboptimal or quickly suppressed by the high evening levels of immunosuppressive factors (Qian et al. 2021; Özdemir et al. 2025). By aligning OV administration with the morning window of peak T-cell effector function and DC alertness, clinicians can exploit a primed immune system to amplify the “vaccine effect” of the virus (Karaboué et al. 2024; Liu et al. 2026). This integration of chrono-immunology into virotherapy protocols, Oncolytic Chronovirotherapy, aims to convert the GBM milieu into a “hot” environment exactly when the host is biologically prepared to sustain a potent anti-tumor attack (Fig. 2).

## Lessons from clinical chronotherapy

### The temozolomide paradigm: From clock genes to patient survival

The most compelling evidence for clinical chronotherapy in neuro-oncology comes from the administration of Temozolomide (TMZ), the standard-of-care DNA alkylating agent for GBM. TMZ is an ideal candidate for chronotherapeutic scheduling due to its rapid absorption, its ability to cross the blood-brain barrier, and its short half-life of approximately 1.8 h (Amiama-Roig et al. 2022; Nelson and Relógio 2024; Nelson et al. 2025). A landmark retrospective study of 166 GBM patients revealed that morning administration of TMZ was associated with a six-month increase in overall survival compared to evening administration, specifically among patients with methylated MGMT (Damato et al. 2021; Gonzalez-Aponte et al. 2026).

**Fig. 3:**
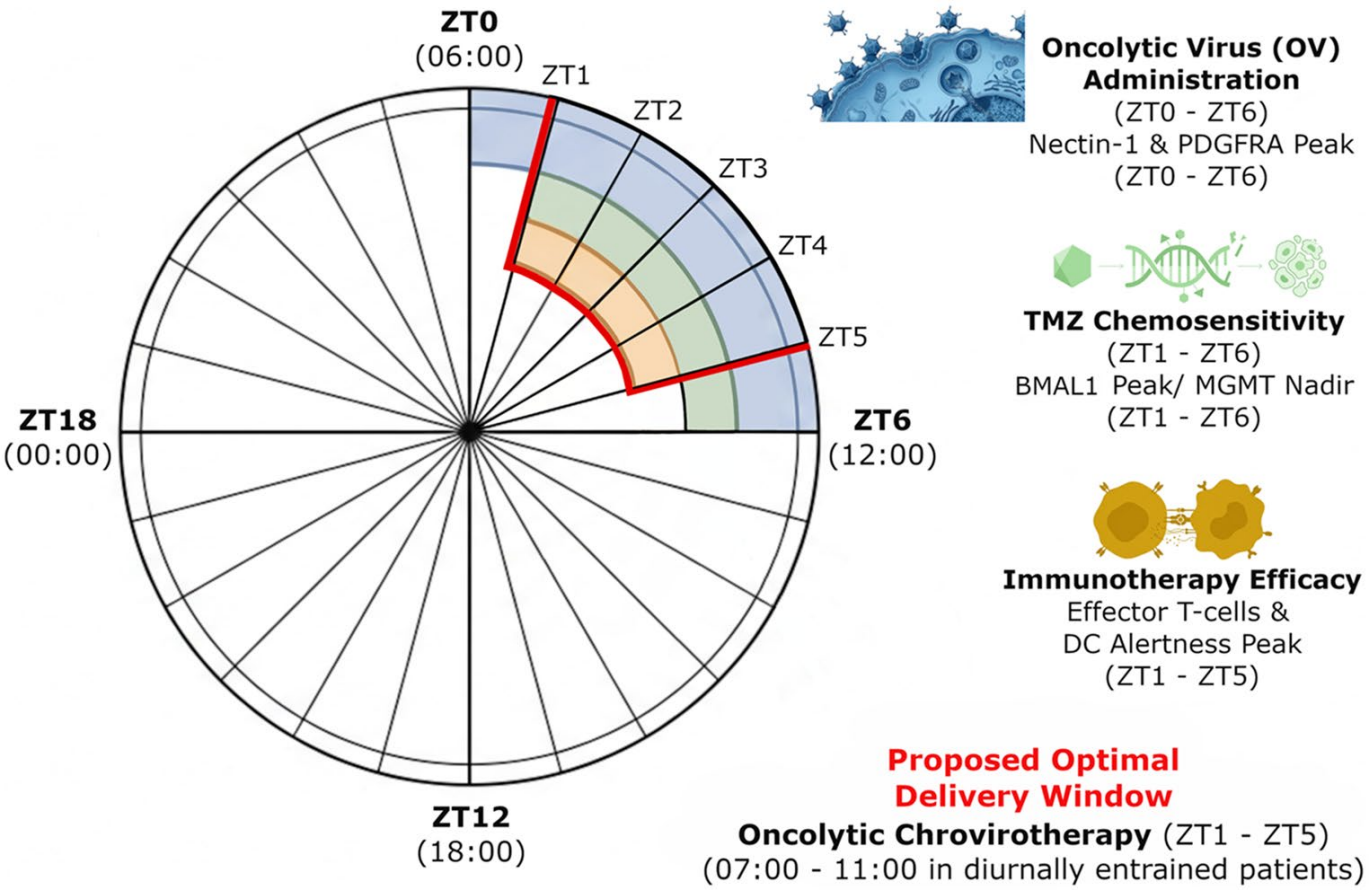
The 24-hour clock diagram illustrates the convergence of three circadian-sensitive therapeutic windows in GBM. The oncolytic virus (OV) administration window (ZT0-ZT6) aligns with the morning peak of Nectin-1 and PDGFRA receptors; the TMZ chemosensitivity window (ZT1-ZT6) coincides with peak BMAL1 expression and the functional nadir of MGMT-mediated DNA repair; and the immunotherapy efficacy window (ZT1-ZT5) reflects peak effector T-cell infiltration and dendritic cell alertness. The overlapping region (ZT1-ZT5, approximately 07:00–11:00 in diurnally entrained patients) constitutes the proposed optimal delivery window for Oncolytic Chronovirotherapy. ZT, Zeitgeber Time; TMZ, temozolomide; MGMT, O6-methylguanine-DNA methyltransferase

This survival benefit is mechanistically anchored in the circadian regulation of the MGMT enzyme. Preclinical models demonstrate that GBM sensitivity to TMZ-induced apoptosis peaks during the daily maximum of BMAL1 expression, which coincides with the nadir of MGMT activity (Albaqami 2025; Nelson et al. 2025; Gonzalez-Aponte et al. 2026). Furthermore, mathematical modeling of MGMT promoter methylation in human biopsies indicates that the probability of detecting methylation peaks around midday, suggesting that the “optimal” diagnostic and therapeutic windows are inherently time-dependent (Nelson et al. 2025; Gonzalez-Aponte et al. 2026) (Fig. 3). However, findings from prospective trials, such as the CENTRIC and CORE trials, have been more controversial, reporting no overall survival benefit but noting increased bone marrow toxicity in the morning group (Nelson et al. 2025). This discrepancy underscores a critical lesson: fixed “wall-clock” timing may fail if the patient’s internal circadian phase is disrupted by the tumor or by the use of dexamethasone, necessitating personalized biological timing rather than rigid scheduling (Nettnin et al. 2025; Nelson et al. 2025).

Dexamethasone (DEX), a ubiquitous corticosteroid in the clinical management of GBM-associated edema, serves as a critical yet complex modulator of the circadian-immune axis. Functioning as a potent synthetic glucocorticoid, DEX acts as a robust ‘zeitgeber’ (time-giver) that can synchronize molecular clocks across tumor and healthy tissues, potentially reinforcing the temporal gating of therapy-sensitive nodes like BMAL1 (Nettnin et al. 2025). However, its chronic administration often results in profound local and systemic immunosuppression that may jeopardize the efficacy of oncolytic chronovirotherapy (Swildens et al. 2022). Specifically, DEX has been shown to counteract the depletion of tumor-associated macrophages (TAMs) induced by oncolytic vectors and to dampen T-cell responsiveness to both immune checkpoint inhibitors and viral-induced antigen presentation (Swildens et al. 2022; Shamekh et al. 2025). Moreover, by suppressing the endogenous hypothalamic-pituitary-adrenal (HPA) axis, DEX can shift the patient’s internal biological phase, causing a mismatch between external ‘wall-clock’ time and the actual windows of tumor vulnerability (Gonzalez-Aponte et al. 2025). Therefore, personalized chronotherapeutic models must integrate individual steroid dosing profiles to ensure that viral delivery coincides with a state of relative immune alertness rather than steroid-induced quiescence.

### Vindicating early infusions: Lessons from melanoma and NSCLC

The “early-is-better” hypothesis has gained massive momentum through the clinical study of Immune Checkpoint Inhibitors (ICIs). The MEMOIR study, a longitudinal analysis of stage IV melanoma patients, demonstrated that receiving at least 20% of ICI infusions (ipilimumab, nivolumab, or pembrolizumab) after 16:30 was independently associated with significantly shorter overall survival (median 4.8 years vs. not reached; HR 2.04) (Qian et al. 2021; Karaboué et al. 2024). This trend is consistent across multiple malignancies; a recent bi-continental study of 713 non-small cell lung cancer (NSCLC) patients receiving immuno-chemotherapy found that an 11:30 cutoff was optimal. Patients treated before 11:30 exhibited nearly double the overall survival (33.0 vs. 19.5 months) and progression-free survival (PFS) compared to those treated in the afternoon (Huang et al. 2025). These outcomes are attributed to the circadian gating of T-cell effector functions and the rhythmic expression of PD-1, which peaks in the morning window of immune “alertness” (Huang et al. 2025; Nagy et al. 2025; Liu et al. 2026). For oncolytic virotherapy, these data suggest that the “vaccine effect” of the virus, triggered by the release of antigens, will likely be most potent if the initial viral “take” and the subsequent immune recruitment occur during these highly responsive morning hours.

### Chrono-radiotherapy: Optimizing the therapeutic index

Radiotherapy, another pillar of GBM treatment, shows a distinct circadian profile primarily related to toxicity management. Studies in head and neck cancer (HNC) demonstrate that morning radiotherapy significantly reduces the risk of severe oral mucositis (Grade ≥ 3) by 31% compared to evening treatment. This is because healthy normal cells are in the G1 phase during the morning, making them more resistant to radiation-induced damage (Abusamak et al. 2025). In GBM specifically, a retrospective review of 109 patients found no significant difference in survival between morning and afternoon radiotherapy (Kisamore et al. 2023; Nelson and Relógio 2024). However, preclinical evidence remains robust, showing that apoptosis induction is highest at the peak of PER2 expression in rat gliomas but not in healthy brain tissue (Nelson and Relógio 2024). The lack of a clear survival benefit in human trials may be due to the fact that high-grade gliomas are profoundly heterogenous and often “dysthymic,” meaning their internal clocks are already too disrupted to respond to a generalized radiation schedule (Amiama-Roig et al. 2022; Abusamak et al. 2025).

### Sex-specific responses and the need for personalization

The EORTC 05963 trial in metastatic colorectal cancer provided a pivotal lesson in chronotherapeutic design (5-FU, oxaliplatin): the same rhythmic infusion that significantly extended survival in men was actually detrimental to women compared to conventional schedules (Karaboué et al. 2024). This sexual dimorphism likely stems from sex-specific variations in the circadian regulation of drug-metabolizing enzymes and the higher amplitude rhythms often observed in females, which can lead to higher toxicities in treatments like 5-FU (Amiama-Roig et al. 2022; Karaboué et al. 2024; Özdemir et al. 2025). Similar trends have been noted in nasopharyngeal carcinoma, where women experience significantly worse adverse events from chemotherapy despite an overall better prognosis than men(Abusamak et al. 2025). Even for modern immunotherapies, patient sex significantly influences drug clearance, suggesting that the ‘optimal’ window for treatment may fundamentally differ between genders (Özdemir et al. 2025).

In the context of glioblastoma, while some preliminary analyses have yet to detect significant sex-based differences in BMAL1 expression patterns, the risk of ‘averaging’ therapeutic responses across genders remains a critical barrier to clinical success (Wan et al. 2025; Nettnin et al. 2025). Ignoring these biological nuances risks the failure of chronotherapeutic protocols by neglecting the distinct temporal vulnerabilities of female patients. Therefore, the path toward a successful ‘oncolytic chronovirotherapy’ must move beyond rigid wall-clock scheduling to incorporate sex-stratified models. This evidence reinforces the central argument of this critical review: that chronotherapy is not merely about finding a universal ‘best’ hour, but about mapping a therapy to the specific biological phase and physiological profile of the individual (Wan et al. 2025; Zhang et al. 2026).

## Perspective: The path to personalized oncolytic chronovirotherapy

The integration of circadian biology into the clinical application of oncolytic viruses (OVs) represents a fundamental paradigm shift from static treatment models to a dynamic, time-aware strategy in neuro-oncology. As highlighted throughout this critical review, the efficacy of viral oncolysis is intrinsically governed by the temporal landscape of both the tumor and the host immune system. We argue that the next generation of glioblastoma (GBM) therapies must move beyond standard “one-size-fits-all” scheduling to adopt Oncolytic Chronovirotherapy, where viral administration is precisely aligned with specific daily windows of maximal cellular vulnerability and peak immune competence.

One of the most formidable challenges to this approach is the profound intratumoral heterogeneity and the “dysthymic” nature of GBM, where the internal clock of malignant cells is often severely dysregulated or subverted to favor growth, stemness, and therapeutic resistance. However, evidence suggests that even in these states of disruption, tumors often remain synchronized with the host’s central pacemaker through systemic signals such as glucocorticoids and autonomic innervation. This residual synchronization implies that the host’s own biological rhythms can serve as accessible proxies for the tumor’s temporal state, allowing clinicians to time viral delivery using personalized circadian biomarkers.

The confirmation of an individual’s chronotype, specifically the “internal time” of the tumor microenvironment, is the cornerstone of clinical success. Emerging technologies, such as multisensory wearable actigraphy monitors, now allow for the continuous, real-time monitoring of rest-activity cycles and physiological parameters like body temperature. When integrated with advanced artificial intelligence algorithms like TimeTeller or BodyTime, which can model the molecular clock from a single transcriptomic profile, these tools provide a comprehensive “intrinsic biological clock map” for each patient. Furthermore, the recent identification of circadian core gene (CCG) patterns offers a robust molecular framework to stratify patients and target biologically vulnerable subgroups, translating circadian-driven susceptibility into measurable survival gains.

The synchronization of OVs with established treatments like temozolomide (TMZ) and radiotherapy offers a unique synergistic opportunity. Retrospective analyses and mathematical models indicate that TMZ sensitivity is maximal when the core-clock protein BMAL1 reaches its daily peak, a phase that coincides with the nadir of the DNA repair enzyme MGMT. By delivering OVs during this early morning window, when viral entry receptors like Nectin-1 are most abundant and dendritic cells are primed for antigen presentation, clinicians can initiate a “coordinated rhythmic attack”. This timing maximizes initial infection and direct lysis while ensuring that the resulting release of DAMPs and tumor-associated antigens occurs when the immune system is most alert and capable of converting a “cold” TME into a “hot,” immune-responsive environment.

These principles extend naturally beyond the HSV-1 scaffold to the full spectrum of OVs that have now established clinical feasibility in GBM (Stavrakaki et al. 2021; Liu et al. 2025). A recent comprehensive review of OV clinical trials in this disease explicitly called for research into the *“optimal dosing window for OVT”* as an unresolved priority (Coates et al. 2024), a question that the Oncolytic Chronovirotherapy framework proposed in this review is designed to address directly. Future trials of DNX-2401, PVSRIPO, MV-CEA, and related platforms should incorporate time-of-day of administration as an explicit, pre-specified randomized variable, informed by the circadian receptor oscillation data outlined herein, to definitively test whether biological timing can translate the preclinical mechanistic evidence into measurable clinical benefit (Zeng et al. 2026).

Looking ahead, the clinical deployment of personalized chronovirotherapy will depend on the development of integrated mechanistic PK-PD models that couple viral replication kinetics with the host’s 24-hour cycle. These models must account for critical variables such as age and sex-specific differences in drug metabolism and immune amplitude, which have already been shown to dictate the success of chronotherapy in other cancers. Future trials should transition to adaptive designs that schedule treatments based on the patient’s individual biological phase rather than external clock time, definitively validating the potential of this temporal frontier.

### Limitations and translational challenges

Despite the compelling biological rationale assembled in this review, several important limitations must be explicitly acknowledged. Most critically, no prospective clinical trial has yet incorporated time-of-day of oncolytic virus administration as a formal, randomized intervention; the chronotherapy efficacy data cited herein derive primarily from retrospective analyses (TMZ, ICIs) and epidemiological studies of other treatment modalities, rather than from OV-specific prospective trials (Qian et al. 2021; Karaboué et al. 2024).

This represents the most fundamental gap between the framework proposed and its clinical validation. The recent review by Coates and collaborates independently identified the *“optimal dosing window”* as an unresolved priority for the OVT field, further underscoring the urgency of addressing this gap in future trial design (Coates et al. 2024). Second, the circadian gating of viral entry receptors, most rigorously documented for Nectin-1 and p75NTR in murine infection models and human cerebral organoids, requires direct validation in resected human GBM tissue, and ideally in intra-tumoral biopsy series collected across multiple circadian time points in situ (Tang et al. 2025; Zeng et al. 2026).

Third, the profoundly “dysthymic” nature of high-grade gliomas, in which the molecular clock of malignant cells may be substantially desynchronized from the host’s systemic rhythms, introduces meaningful uncertainty into the assumption that wearable-derived patient chronotypes reliably predict intra-tumoral receptor oscillation timing (Petkovic et al. 2023; Abusamak et al. 2025). Fourth, the practical implementation of morning-specific OV delivery within real-world neurosurgical infrastructure, where scheduling is governed by operating room availability, anesthesia logistics, recovery capacity, and patient transfer constraints, presents considerable operational challenges that clinical protocols must explicitly address and pilot-test (Kisamore et al. 2023; El-Tanani et al. 2024).

Finally, individual variability in chronotype, corticosteroid dosing, sex-specific clock amplitude, and tumor burden will necessitate personalized rather than population-level timing prescriptions, adding complexity to trial design (Lévi et al. 2024; Gonzalez-Aponte et al. 2025, 2026). These limitations underscore that the transition from biological framework to clinical protocol will require carefully designed, prospective, adaptive trials in which time-of-day is treated as an explicit, independently randomized variable.

## Conclusion

Oncolytic Chronovirotherapy addresses the critical “fourth dimension” of cancer therapy: time. By leveraging the evolutionary mechanisms that neurotropic viruses exploit to hijack host circadian rhythms, and by utilizing modern diagnostic tools to map individual circadian phases, we may ultimately be positioned to overcome the formidable resistance that has limited glioblastoma treatment for decades. The transition from static, clock-agnostic interventions to dynamic, chronomodulated therapeutic regimes represents a necessary evolution in precision neuro-oncology. As wearable technologies and artificial intelligence-driven clock inference methods mature, the integration of personalized circadian profiling into clinical trial design will become increasingly feasible. Ultimately, respecting the temporal biology of both tumor and host, not fighting against it, offers a biologically grounded path toward what may ultimately become a more effective strategy for oncolytic viruses and, more broadly, transforming the therapeutic landscape of glioblastoma.

## Data Availability

No datasets were generated or analysed during the current study.
